# TERT-Promoter Mutational Status in Glioblastoma – Is There an Association With Amino Acid Uptake on Dynamic ^18^F-FET PET?

**DOI:** 10.3389/fonc.2021.645316

**Published:** 2021-04-27

**Authors:** Marcus Unterrainer, Viktoria Ruf, Katharina von Rohr, Bogdana Suchorska, Lena Maria Mittlmeier, Leonie Beyer, Matthias Brendel, Vera Wenter, Wolfgang G. Kunz, Peter Bartenstein, Jochen Herms, Maximilian Niyazi, Jörg C. Tonn, Nathalie Lisa Albert

**Affiliations:** ^1^ Department of Radiology, University Hospital, LMU Munich, Munich, Germany; ^2^ Department of Nuclear Medicine, University Hospital, LMU Munich, Munich, Germany; ^3^ German Cancer Consortium (DKTK), Partner Site Munich and German Cancer Research Center (DKFZ), Heidelberg, Germany; ^4^ Department of Neuropathology and Prion Research, LMU Munich, Munich, Germany; ^5^ Department of Neurosurgery, University Hospital, LMU Munich, Munich, Germany; ^6^ Department of Radiation Oncology, University Hospital, LMU Munich, Munich, Germany

**Keywords:** amino acid PET, molecular genetics, glioblastoma, TERT (telomerase reverse transcriptase), FET PET

## Abstract

**Objective:**

The mutation of the ‘telomerase reverse transcriptase gene promoter’ (TERTp) has been identified as an important factor for individual prognostication and tumorigenesis and will be implemented in upcoming glioma classifications. Uptake characteristics on dynamic ^18^F-FET PET have been shown to serve as additional imaging biomarker for prognosis. However, data on the correlation of TERTp-mutational status and amino acid uptake on dynamic ^18^F-FET PET are missing. Therefore, we aimed to analyze whether static and dynamic ^18^F-FET PET parameters are associated with the TERTp-mutational status in de-novo *IDH*-wildtype glioblastoma and whether a TERTp-mutation can be predicted by dynamic ^18^F-FET PET.

**Methods:**

Patients with de-novo *IDH*-wildtype glioblastoma, WHO grade IV, available TERTp-mutational status and dynamic ^18^F-FET PET scan prior to any therapy were included. Here, established clinical parameters maximal and mean tumor-to-background-ratios (TBR_max_/TBR_mean_), the biological-tumor-volume (BTV) and minimal-time-to-peak (TTP_min_) on dynamic PET were analyzed and correlated with the TERTp-mutational status.

**Results:**

One hundred *IDH*-wildtype glioblastoma patients were evaluated; 85/100 of the analyzed tumors showed a TERTp-mutation (C228T or C250T), 15/100 were classified as TERTp-wildtype. None of the static PET parameters was associated with the TERTp-mutational status (median TBR_max_ 3.41 vs. 3.32 (p=0.362), TBR_mean_ 2.09 vs. 2.02 (p=0.349) and BTV 26.1 vs. 22.4 ml (p=0.377)). Also, the dynamic PET parameter TTP_min_ did not differ in both groups (12.5 vs. 12.5 min, p=0.411). Within the TERTp-mutant subgroups (i.e., C228T (n=23) & C250T (n=62)), the median TBR_max_ (3.33 vs. 3.69, p=0.095), TBR_mean_ (2.08 vs. 2.09, p=0.352), BTV (25.4 vs. 30.0 ml, p=0.130) and TTP_min_ (12.5 vs. 12.5 min, p=0.190) were comparable, too.

**Conclusion:**

Uptake characteristics on dynamic ^18^F-FET PET are not associated with the TERTp-mutational status in glioblastoma However, as both, dynamic ^18^F-FET PET parameters as well as the TERTp-mutation status are well-known prognostic biomarkers, future studies should investigate the complementary and independent prognostic value of both factors in order to further stratify patients into risk groups.

## Introduction

According to the updated 2016 WHO classification of brain tumors, the molecular genetic profile plays a major role for the glioma characterization and highly affects the further clinical management and treatment strategies (1, 2). Beyond the current molecular genetic stratification using the isocitrate dehydrogenase (*IDH*)-mutational status and 1p/19q-codeletion, additional molecular genetic markers are increasingly identified and gradually gain access into clinical routine. In particular, mutations of the telomerase reverse transcriptase gene promoter (TERTp) were identified as important factor within the tumorigenesis and individual prognostication (3, 4), with inferior outcome in combination with an *IDH*-wildtype status (5, 6), which will be implemented in upcoming glioma classifications.

As recommended by the Response assessment in Neurooncology (RANO) working group in their clinical guidelines (7–9), molecular imaging using positron-emission-tomography (PET) with radiolabeled amino acids such as O-(2-^18^F-fluoroethyl)-L-tyrosine (^18^F-FET) is increasingly used for the comprehensive evaluation and characterization of brain neoplasms beyond morphological standard imaging with MRI, e. g. for treatment planning (10–13), but also for noninvasive tumor characterization at initial diagnosis (14–20). Recent studies indicated that the *IDH*-mutational status is highly associated with ^18^F-FET PET uptake in brain tumors, especially with the ‘minimal time to peak’ (TTP_min_) on dynamic ^18^F-FET PET, and has thus a high diagnostic power for the identification of *IDH*-wildtype gliomas (21). With regard to TERTp, no study has hitherto evaluated the association of amino acid uptake on PET and the TERTp-mutational status. Hence, we aimed to assess whether the uptake characteristics on static and dynamic ^18^F-FET PET are likewise associated with the TERTp-mutation status in a homogeneous group of de-novo, *IDH*-wildtype glioblastoma and whether PET can predict the TERTp-mutation status.

## Methods and Materials

### Patients

Patients with histologically confirmed, newly diagnosed *IDH*-wildtype glioblastoma WHO grade IV with available molecular genetic analyses of the TERT-promoter mutation status as well as a dynamic ^18^F-FET PET scan prior to stereotactic biopsy or surgical resection were identified. All patients have given written informed consent prior to the PET examination as part of the clinical routine. Ethical approval of the retrospective study protocol was given by the institutional review board of the LMU.

### Histological Confirmation, Tumor Grading and Molecular Genetic Analysis

Stereotactic biopsy procedures and microsurgical resections were performed at the Department of Neurosurgery, LMU Munich, Germany. As part of the clinical routine, histopathological and molecular genetic evaluations were performed at the Institute of Neuropathology, LMU Munich, Germany, and were initially classified according to the 2007 WHO classification of brain tumors (22) and were re-classified according to the updated 2016 WHO classification (1). The *IDH*-mutation status and TERT-promoter methylation were analyzed in clinical routine according to standard protocols (23–25). For further specification regarding the histopathological workup see also (26, 27).

### ^18^F-FET PET Image Acquisitionand Data Analysis

^18^F-FET PET scans were performed at the Department of Nuclear Medicine, LMU. Data of the dynamic ^18^F-FET PET scans were acquired using an ECAT Exact HR+ scanner (Siemens). After a 15-min transmission scan with a ^68^Ge rotating rod source, approximately 180 MBq of ^18^F-FET were injected. Dynamic emission recording was accomplished after tracer injection up to 40 min post injection in 3-D mode consisting of 16 frames (7 x 10 s; 3 x 30 s; 1 x 2 min; 3 x 5 min; 2 x 10 min). Two-dimensional filtered back-projection, reconstruction algorithms using a 5 mm Hann Filter were used for image reconstruction and corrected for photon attenuation and model-based scatter. The mean background activity (BG) was assessed using 6 large crescent-shaped regions of interests (ROI) in the frontal lobe of the healthy contralateral hemisphere fused to a volume of interest (VOI), in which the mean BG was derived (28). The biological tumor volume (BTV) was estimated by a semiautomatic threshold-based delineation of a volume of interest (VOI) using a standardized uptake value (SUV) threshold of 1.6 x BG, as previously described as optimal threshold (29). The maximal SUV (SUV_max_) and mean SUV (SUV_mean_) as derived within the BTV were then divided by the BG resulting in mean and maximal tumor-to-background ratio (TBR_mean_/TBR_max_). Data on dynamic PET was evaluated using the software PET Display Dynamic implemented in the Hermes workstation: in early summation images (10-30 min p.i.), a 90% isocontour region of interest was created to extract the time-activity-curves (TACs) on a slice-by-slice manner. Then, the time to peak (TTP) was assessed on each slice of the tumor and the shortest TTP in at least 2 consecutive slices was defined as minimal TTP (TTP_min_), see also (30, 31).

### Statistics

SPSS for Windows (version 23.0; SPSS, Chicago, IL) was used for statistical analyses. Descriptive statistics are displayed as median (range). Normal distribution was assessed using the Shapiro-Wilk-test. The unpaired Wilcoxon-test was used for independent and not-normally distributed continuous parameters. Receiver operating curves (ROC) analyses were used to assess the diagnostic power of continuous parameters, the ‘Area under the curve’ (AUC) served as quantitative measure for the diagnostic power. Statistical significance was defined as a two-tailed p-value <0.05.

## Results

### Patients

One-hundred patients with de-novo, *IDH*-wildtype glioblastoma, WHO grade IV were included (62/100 (62.0%) male, 38/100 (38.0%) female). The median age was 62.0 (range, 30.1-82.7) years. Tissue samples for histological and molecular genetic analyses were obtained by stereotactic biopsy in 74/100 (74.0%) and by surgical resection in 26/100 (26.0%) cases. Overall, 15/100 (15%) did not comprise a TERTp-mutation and were classified as TERTp-wildtype. Of the remaining 85/100 (85%) patients with TERTp-mutation, 62/85 (72.9%) showed a C228T-mutation and 23/85 (27.1%) showed a C250T-mutation.

### Overall ^18^F-FET-Uptake Characteristics

All included gliomas were ^18^F-FET-positive providing a median TBR_max_ of 3.37 (2.06-7.07), a median TBR_mean_ of 2.06 (range, 1.70-2.92) and a median BTV of 25.8 (range, 3.8-133.3) ml. In the dynamic analysis, median TTP_min_ was 12.5 (range, 3.0-35.0) minutes with a small proportion of late TTP_min_ ≥ 25 minutes in 13/100 (13.0%) cases only.

### ^18^F-FET-Uptake Characteristics Comparing TERTp-Mutant and TERTp-Wildtype Glioblastomas and Predictability of TERTp Mutational Status

**Figure 1:**
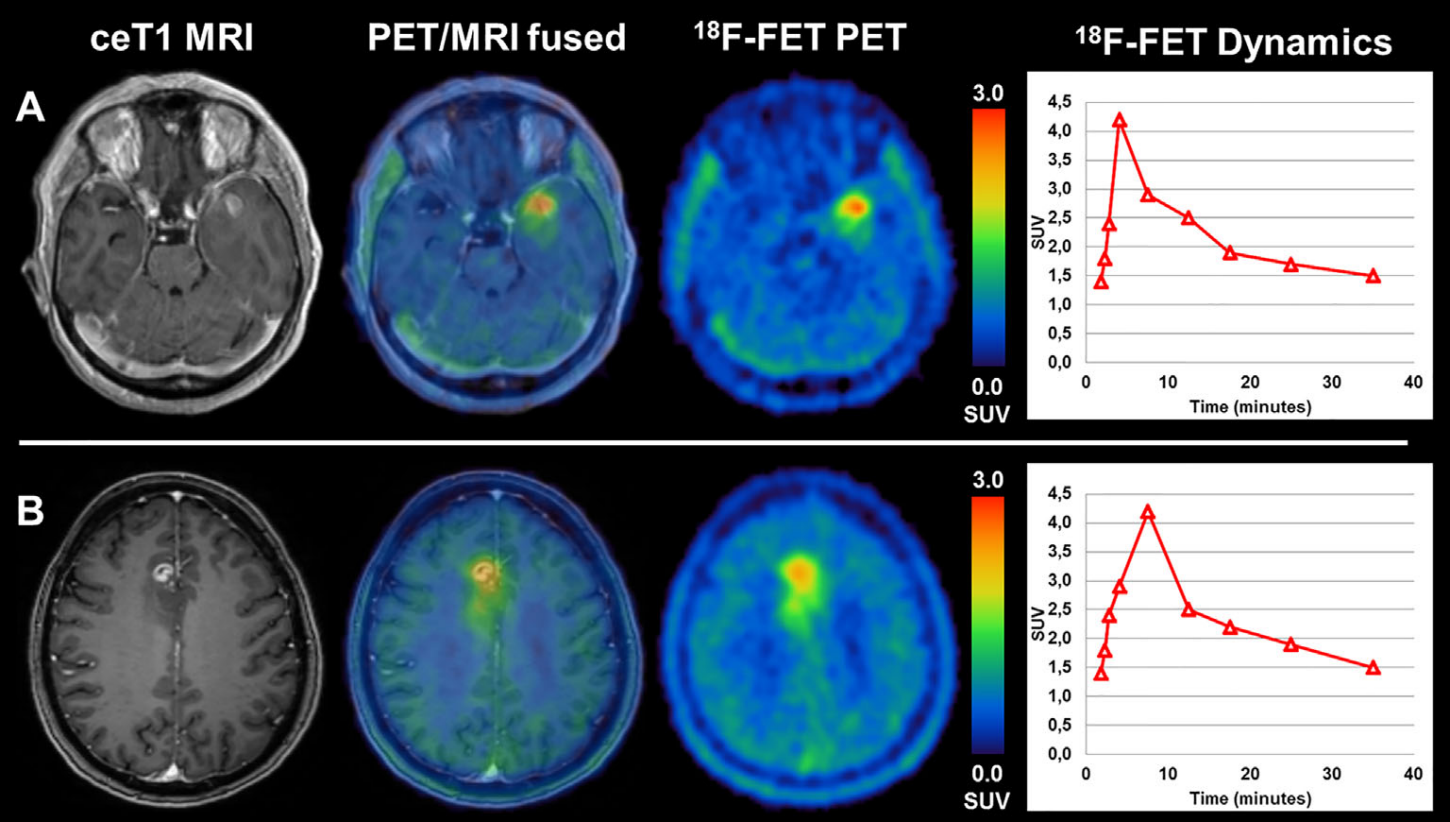
**(A)** a patient with TERTp-mutant glioblastoma (TBR_max_ 4,1; TBR_mean_ 2,3; BTV 15,4 ml, TTP_min_ 5_ min_) shows comparable, only slightly diverging imaging features as **(B)**, a patient with TERTp-wildtype glioblastoma (TBR_max_ 2,9; TBR_mean_ 1,9; BTV 12,3 ml, TTP_min_ 10_ min_).

Comparing glioblastomas with TERTp-mutation (n=85) and those without (n=15) revealed no statistically significant difference in terms of median TBR_max_ (3.41 (range, 2.06-7.07) vs. 3.32 (range, 2.32-4.67), p=0.362), TBR_mean_ (2.09 (range, 1.70-2.92) vs. 2.02 (range, 1.79-2.56), p=0.349) and BTV (26.1 (range, 3.8-133.3) ml vs. 22.4 (range, 3.9-75.7) ml, p=0.377). Not only the evaluated static PET parameters, but also the dynamic parameter TTP_min_ did not differ between those two groups (12.5 (range, 3.0-35.0) min vs. 12.5 (range, 7.5-25.0) min, p=0.411). By consequence, the ROC-analysis to assess the diagnostic power of ^18^F-FET PET for the prediction of the TERTp mutational status did not reveal reliable thresholds for the differentiation between TERTp-mutant and TERTp-wildtype glioblastomas. Analyzing the static parameters TBR_max_, TBR_mean_ and BTV, the AUC ranged between 0.572 and 0.576 only. Also, for the dynamic parameter TTP_min_ the AUC reached only 0.562 at a best cut-off at 7.5 min. Further specifications can be found in **Table 1** and **Table 2**. Patient examples can be found on **Figure 1**.

**Table 1:** Influence of TERT-mutation on ^18^F-FET-uptake characteristics [median (range)].

	Overall (n=100)	TERT-mutation (n=85)	TERT-wildtype (n=15)	Level of significance
**TBR_max_**	3.37 (2.06-7.07)	3.41 (2.06-7.07)	3.32 (2.32-4.67)	p=0.362
**TBR_mean_**	2.06 (1.70-2.92)	2.09 (1.70-2.92)	2.02 (1.79-2.56)	p=0.349
**BTV**	25.8 (3.8-133.3) ml	26.1 (3.8-133.3) ml	22.4 (3.9-75.7) ml	p=0.377
**TTP_min_**	12.5 (3.0-35.0) min	12.5 (3.0-35.0) min	12.5 (7.5-25.0) min	p=0.411

**Table 2:** Diagnostic power of ^18^F-FET PET for detection TERTp mutation.

Parameter	Best cut-off value	Area under the curve	Level of significance
**TBR_max_**	3.60	0.574	p=0.323
**TBR_mean_**	2.21	0.576	p=0.297
**BTV**	24.1 ml	0.572	p=0.359
**TTP_min_**	7.5 min	0.562	p=0.391

### ^18^F-FET-Uptake Characteristics Comparing TERT-Mutation Subtypes (C228T vs. C250T)

Comparing the two subtypes of TERT-promoter mutation C228T (n=62) & C250T (n=23), there was also no statistically significant difference in terms of median TBR_max_ (3.33 (range, 2.06-5.51) vs. 3.69 (range, 2.37-7.07), p=0.095), TBR_mean_ (2.08 (range, 1.70-2.56) vs. 2.09 (range, 1.79-2.92), p=0.352) or BTV (25.4 (range, 3.8-133.3) ml vs. 30.0 (range, 5.7-102.1) ml, p=0.130). On dynamic PET analyses, the median TTP_min_ was also statistically comparable between those two mutation subtypes (12.5 (range, 7.5-35.0) min vs. 12.5 (range, 3.0-35.0) min, p=0.190). For further specifications, please see **Table 3**.

**Table 3:** Influence of TERT-mutation subtypes on ^18^F-FET-uptake characteristics [median (range)].

	TERT-mutation overall (n = 85)	C228T (n = 62)	C250T (n = 23)	Level of significance
**TBR_max_**	3.41 (2.06-7.07)	3.33 (2.06-5.51)	3.69 (2.37-7.07)	p=0.095
**TBR_mean_**	2.09 (1.70-2.92)	2.08 (1.70-2.56)	2.09 (1.79-2.92)	p=0.352
**BTV**	26.1 (3.8-133.3) ml	25.4 (3.8-133.3) ml	30.0 (5.7-102.1) ml	p=0.130
**TTP_min_**	12.5 (3.0-35.0) min	12.5 (7.5-35.0) min	12.5 (3.0-35.0) min	p=0.190

## Discussion

This is the first study evaluating the association of amino acid uptake by means of ^18^F-FET PET and the TERTp-mutational status in glioma patients. As the TERTp-mutational status has shown additional prognostic value in *IDH*-wildtype gliomas/glioblastomas (5, 6, 32, 33), a non-invasive tool for the prediction of a TERTp-mutation would be helpful for the clinical management of glioma patients. In our large cohort with homogeneous histological and molecular genetic profile (i.e. WHO grade IV glioblastoma, *IDH*-wildtype only), we observed a high proportion of patients with TERTp mutation of 85%, which is in line with the proportion of patients with TERTp mutation in the current literature (4). Moreover, within the group of TERTp mutant glioblastomas, the C228T-mutation was present more frequently (72.9%) than the C250T-mutation (27.1%), which is also in line with the distribution within *IDH*-wildtype glioblastomas as described in the current literature (6, 34).

Comparing the TERTp-mutational status with the static PET parameters in terms of uptake intensity (TBR_max_ and TBR_mean_) and tumor extent (BTV), we observed a high overlap between TERTp-mutant and TERTp-wildtype tumors so that no cutoff could be found to differentiate between those groups. Moreover, TTP_min_ on dynamic PET was also indifferent between TERTp-mutant and TERTp-wildtype glioblastomas. Taken together, both groups presented with comparable imaging findings and could not be distinguished on ^18^F-FET PET. This leads to the assumption that dynamic ^18^F-FET PET cannot predict the TERTp-mutational status in *IDH*-wildtype glioblastoma. Taking a closer look at the *IDH*-mutational status, however, recent studies indicated that the *IDH*-mutational status can be identified non-invasively by dynamic ^18^F-FET PET with a relatively high diagnostic accuracy. In particular, the prognostically poor *IDH*-wildtype status can be predicted by a short TTP_min_ on dynamic ^18^F-FET PET (21).

When analyzing the TERTp-mutation subtypes (i.e. C228T & C250T), expectedly, no difference in terms of uptake-intensity (TBR_max_ & TBR_mean_) and tumor extent (i.e. BTV) on PET could be observed; also, TTP_min_ on dynamic PET was indifferent between C228T & C250T mutations. This finding, however, is not surprising, as these two mutations of hot spot promoter regions (C228T and C250T) are basically responsible for the same molecular mechanism (32, 33).

In general, one could speculate that the pathophysiological changes that are accompanied with TERTp mutations and their influence on cell regulation might also affect the cellular metabolism in terms of amino acid metabolism. Moreover, one could argue that both static and dynamic ^18^F-FET PET parameters were described to be of prognostic value in the further disease course of glioma patients; as the same is true for TERTp mutations, a certain intercorrelation does not seem unlikely. On a molecular level, the TERT as a catalytic subunit of the telomerase enzyme complex is critically involved in telomere maintenance and lengthening. Abnormal upregulation and activity of TERT as a consequence of TERTp-mutations are considered one of the mechanisms of cellular immortality in cancer cells during division, particularly in gliomas (35–37). With regard to PET imaging, the activity and/or expression of the large neutral amino acid transporter (LAT) at the tumor cells and at the brain capillary endothelial cells (38) is considered a key factor responsible for the intracellular uptake of amino acids in gliomas (39). The very exact mechanisms and the histopathological or even molecular genetic correlate resulting in diverging uptake dynamics of ^18^F-FET are not fully clarified yet and may be influenced by further factors such as vascularization. Our study findings suggest that the presence or, vice versa, the absence of TERTp-mutation in glioblastoma and the accompanying features on a cellular basis, although prognostically relevant, do neither result in an altered level of amino acid metabolism nor in changes of uptake dynamics on ^18^F-FET PET.

Notably, there is an occurrence of TERTp-mutations in different tumor types as well, also in molecular subgroups with superior prognosis compared to *IDH*-wildtype gliomas, e. g. in *IDH*-mutant gliomas. Interestingly, among *IDH*-mutant gliomas, *IDH*-mutant gliomas with TERTp-mutation comprise a superior clinical outcome compared to *IDH*-mutant glioma without TERTp-mutation, also with emphasis on the particular histological features (5). Therefore, the presence of TERTp-mutations in brain tumors per se is not necessarily linked to a more aggressive course in general. Particularly, in the group of oligodendroglial tumors (i.e. gliomas with both *IDH*-mutation and 1p/19q-codeletion), basically every tumor presents with TERTp-mutation. This molecular genetic subgroup is associated with favorable outcome compared to e.g. *IDH*-wildtype gliomas (32, 40), despite a basically general presence of TERTp mutations. These phenomena also warrant further investigation of PET-based imaging characteristics in the subgroup of *IDH*-mutant gliomas. First preliminary data using radiomic features on MRI could show certain moderate diagnostic power for the detection of TERTp mutations particularly in low-grade/*IDH*-mutant gliomas (41, 42).

Moreover, a vast body of literature exists dealing with radiomics, deep learning and machine learning with special emphasis on (^18^F-FET) PET and hybrid imaging in neuro-oncology (43–49), not just for the differentiation of treatment-related changes from real progression (44, 50, 51), but also for the predication of prognostically relevant mutations such as the *IDH*-mutation (52). Hence, it needs to be evaluated, if further PET-based analyses with the extraction of radiomic features may add value to the conventional image analysis in order to non-invasively identify the TERTp-mutational status. Interestingly, the predictability of key mutations using standard and advanced PET quantification also seems to vary depending on the used radioligands (53–57).

Particularly, as dynamic ^18^F-FET PET was previously reported to show a high prognostic value in gliomas in addition to the clinically most important molecular genetic biomarkers according to the 2016 WHO classification [*IDH*-mutation and 1p/19q-codeletion status (14, 26)], it would be interesting to evaluate whether the additional prognostic value of PET remains even after further subgroup stratification according to the TERTp mutation status. In order to test this hypothesis, further studies with a larger number of patients (particularly in the relatively small TERTp-wildtype subgroup) are needed to perform multivariate analyses.

Limitations arise from the retrospective study design. Moreover, as mentioned above, the absolute number of tumors without TERTp mutations is relatively low (i.e. n=15 vs. n=85), however, this proportion is in line with the previously reported distribution of TERTp mutations in glioblastoma. In the current manuscript, only filtered-back projection (FBP) reconstructions were used due to the applied scanner; quantification of PET parameters could potentially be diverging using other reconstruction algorithms such as ordered subset expectation maximization (OSEM).

## Conclusion

The prognostically relevant TERTp-mutational status in *IDH*-wildtype glioblastoma is not associated with uptake characteristics on dynamic ^18^F-FET PET. As both, dynamic ^18^F-FET PET parameters as well as the TERTp-mutation status are well-known prognostic biomarkers, but show no association in our analysis, it seems highly interesting to evaluate in larger studies if both factors are independent predictors of patients’ survival and can thereby further stratify patients into risk groups.

## Data Availability Statement

The raw data supporting the conclusions of this article will be made available by the authors upon reasonable request.

## Ethics Statement

The studies involving human participants were reviewed and approved by Ethics committee, LMU Munich. Written informed consent for participation was not required for this study in accordance with the national legislation and the institutional requirements.

## Author Contributions

Conceptualization, MU and NA. Methodology, MU, VR, KR, BS, LM, LB, MB, VW, and WK. Formal analysis, MU, VR, KR, and NA. Resources, all authors. Writing—original draft preparation, MU and NA. Writing—review and editing, all authors. Visualization, MU and NA. Supervision, NA, MN, JH, JT, and PB. Project administration, MU and NA. All authors contributed to the article and approved the submitted version.

## Conflict of Interest

NA is a member of the Neuroimaging Committee of the EANM.

The remaining authors declare that the research was conducted in the absence of any commercial or financial relationships that could be construed as a potential conflict of interest.
